# Comprehensive characterization of an individual’s LINE-1 insertion profile using next generation sequencing data

**DOI:** 10.1186/1471-2105-13-S12-A2

**Published:** 2012-07-31

**Authors:** Alexander Kemper, Musa Hindi, Kenneth S Ramos, Theodore S Kalbfleisch

**Affiliations:** 1 Department of Biochemistry and Molecular Biology, University of Louisville, Louisville, KY 40292, USA; 2 Department of Computer Engineering and Computer Science, University of Louisville, Louisville, KY 40292, USA

## Background

The Long Interspersed Nuclear Element-1 (LINE-1) is an active retrotransposon capable of affecting cellular phenotype in terms of repressing or stimulating the expression of neighboring genes, and even modifying genetic structure via retrotransposition. New LINE-1 insertion events are occurring constantly in somatic cells. They also occur in germline cells resulting in an estimated 1 new LINE-1 element for every 30 live births. As a result of this activity, in each individual, there are estimated to be several thousand LINE-1 elements, with on the order of 1/3 of these elements differing in genotype, homozygous present, homozygous absent, or heterozygous between any two individuals. Given its potential impact on cellular phenotype and function, it is essential to fully characterize an individual’s LINE-1 insertion profile in terms of number of insertion sites, and the sequence and genotype of each insertion.

## Results

By using a next generation sequencing protocol that sequences the ends of genomic DNA fragments 5000 bases in length, we are developing a methodology that can be used to both sequence an individual’s genome, and comprehensively characterize that individual’s LINE-1 insertion profile. We have developed a workflow that will both map fragments to the reference genome for standard variant analysis, and isolate those sequence reads that provide sequence data for the multitude of LINE-1 insertions in the individual’s genome. Once isolated, we are able to assemble the reads and build contigs that allow us to measure the genotype, and predict the phenotypic activity of each LINE-1 element in an individual’s genome. Our method is price competitive with the sequencing of smaller fragments, and provides much more comprehensive information.

## Conclusion

We are developing a method that will allow researchers and clinicians to comprehensively characterize the LINE-1 retrotransposon insertion profile in individuals. Current Next Generation Sequence mapping algorithms will neither identify non-reference LINE-1s, nor will they provide LINE-1 genotypes resulting from an individual’s whole genome sequence analysis. Since LINE-1 elements can greatly impact cellular function and human health, it is essential that we incorporate algorithms such as this to characterize an individual’s LINE-1 insertion profile as we endeavor use genetic information in the treatment of patients.

